# Prediction of hepatitis C virus interferon/ribavirin therapy outcome based on viral nucleotide attributes using machine learning algorithms

**DOI:** 10.1186/1756-0500-7-565

**Published:** 2014-08-23

**Authors:** Amir Hossein KayvanJoo, Mansour Ebrahimi, Gholamreza Haqshenas

**Affiliations:** Department of Biology, School of Basic Sciences, University of Qom, Qom, Iran; Microbiology Department, Monash University, Melbourne, Australia

## Abstract

**Background:**

Hepatitis C virus (HCV) causes chronic hepatitis C in 2-3% of world population and remains one of the health threatening human viruses, worldwide. In the absence of an effective vaccine, therapeutic approach is the only option to combat hepatitis C. Interferon-alpha (IFN-alpha) and ribavirin (RBV) combination alone or in combination with recently introduced new direct-acting antivirals (DAA) is used to treat patients infected with HCV. The present study utilized feature selection methods (Gini Index, Chi Squared and machine learning algorithms) and other bioinformatics tools to identify genetic determinants of therapy outcome within the entire HCV nucleotide sequence.

**Results:**

Using combination of several algorithms, the present study performed a comprehensive bioinformatics analysis and identified several nucleotide attributes within the full-length nucleotide sequences of HCV subtypes 1a and 1b that correlated with treatment outcome. Feature selection algorithms identified several nucleotide features (e.g. count of hydrogen and CG). Combination of algorithms utilized the selected nucleotide attributes and predicted HCV subtypes 1a and 1b therapy responders from non-responders with an accuracy of 75.00% and 85.00%, respectively. In addition, therapy responders and relapsers were categorized with an accuracy of 82.50% and 84.17%, respectively. Based on the identified attributes, decision trees were induced to differentiate different therapy response groups.

**Conclusions:**

The present study identified new genetic markers that potentially impact the outcome of hepatitis C treatment. In addition, the results suggest new viral genomic attributes that might influence the outcome of IFN-mediated immune response to HCV infection.

**Electronic supplementary material:**

The online version of this article (doi:10.1186/1756-0500-7-565) contains supplementary material, which is available to authorized users.

## Background

Hepatitis C virus (HCV) is a blood-borne virus, which causes chronic hepatitis in humans. Despite its discovery over 2 decades ago [1], HCV remains one of the major health threatening infectious agents worldwide. Recent estimations indicate that approximately 2-3% of world population (125–175 million) suffer from chronic hepatitis C [2]. So far, at least six major HCV genotypes (1–6) with less than 72% nucleotide identities, each comprised of several subtypes (1a, 1b, etc.) with 75-86% nucleotide identities, have been identified. The single-stranded viral RNA genome with a size of ~9.6 kb replicates through a double-stranded intermediate form. High frequency of point mutations in the HCV genome during virus replication and the virion structure [3] are major factors hindering the development of a preventive vaccine. To identify an effective therapeutic approach, HCV biology and viral structural (core, E1, and E2) and non-structural (NS) (NS2-3, NS4A-B, NS5A-B) proteins have been extensively studied. Currently, therapeutic regimens for treatment of HCV–infected patients involve HCV direct-/indirect-acting antivirals. Combination of pegylated interferon-alpha (IFN-alpha) and ribavirin (RBV) is prescribed by physicians for treatment of hepatitis C. IFN, a known broadly acting antiviral cytokine, is an essential component of innate immune response. The exact mechanism of action of RBV remains unknown although it improves response rate when it is combined with interferon [4]. The recently FDA approved direct-acting antivirals (DAA, telaprevir and boceprevir) that are used in combination with IFN/RBV have improved HCV therapy success rate by 16-40% [5, 6]. Long-term IFN/RBV combination treatment (24–48 weeks) is required to achieve sustained virological response (SVR). Some patients resolve the virus at the completion of treatment (responders), of whom a proportion demonstrate a virus rebound within 6 months post-treatment (relapsers). Some HCV patients are resistant to combination therapy (non-responders). The success rate of HCV treatment depends on many host and viral factors. Patients who are chronically infected with HCV genotype 1 poorly respond to the combination treatment (about 50% SVR) while higher response rate is observed when patients are infected with genotypes 2 and 3 (about 70-80% SVR). The genotype-dependent therapy response rate suggests that the composition of viral nucleotide and amino acid sequences may impact the therapy outcome. Several comparative analyses have indicated that the amino acid sequences of the HCV proteins including core [7, 8], E2 [9–13], p7 [13], NS2 [13, 14], NS5A [10–13, 15] and NS5B [16] may the success rate of the combination treatment. In addition, host parameters including genetic polymorphism in IL28B locus have been indicated as therapy response rate determinants [17, 18].

There is insufficient data describing nucleotide attributes that correlate with response to therapy. In addition, genomic determinants that may predict the relapse of the disease following a successful clearance remain unclear. This study aims to use various clustering, screening, and decision tree models to analyse full-length HCV genomes and identify novel genetic markers for the prediction of HCV therapy outcome.

## Results

**Figure 1:**
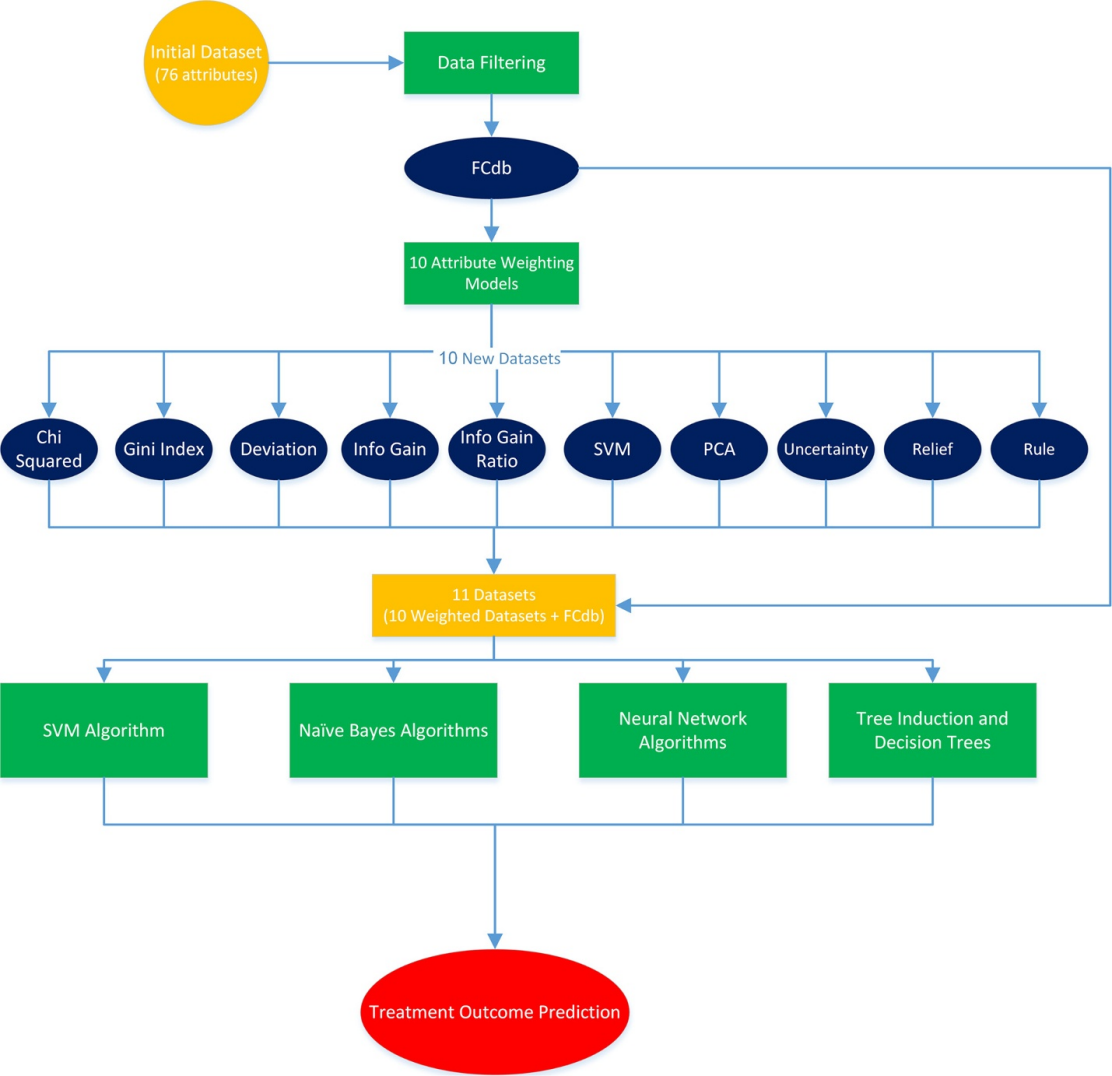
**Flowchart of data-mining processes that were applied to each of comparative groups.**

The initial dataset contained 93 full-length nucleotide sequences of HCV subtypes 1a and 1b from Virahep study [19]. A summary of all data processing steps adopted in this study to predict therapy outcome has been presented in Figure 1. For each sequence, 76 gene attributes were computed. Using data filtering algorithms, useless and closely related attributes (correlations higher than 95%) were excluded. Overall, 35–37 attributes were identified useful for the identification of different treatment outcome groups.

### Attribute weighting

The importance and the contribution of each useful attribute in building the target variable (response to treatment) was evaluated by attribute weighting algorithms. Data was normalized to give a value between 0 and 1 to each weight [20–23]. The important attributes and the number of models which allocated a weight above 0.5 to each attribute have been presented in Table 1A and B, respectively. All Attributes and the relevant weighting models have been presented in an Additional file (see Additional file 1). Only attributes that gained weights higher than 0.5 were used by prediction and tree induction algorithms to predict the response to treatment.

**Table 1:** **Most important nucleotide attributes that were selected by different weighting algorithms**

A			
Subtype 1a (Responders vs.		Subtype 1b (Responders vs.	
Non-Responders)		Non-Responders)	
Attribute	No. of selective	Attribute	No. of selective
	attribute		attribute
	weightings		weightings
	(out of 10)		(out of 10)
Count of hydrogen	9	Count of GC	8
Count of oxygen	8	**Count of UA**	7
Count of CA	7	DS Count of nitrogen	7
Count of CG	7	Count of AU	6
Count of Cytosine	7	Count of GG	5
Count of Guanine	7	Count of Uracil	5
Count of GU	6		
Count of UU	5		
**Count of UA**	5		
Count of CC	5		
**B**			
**Subtype 1a (Responders vs.**		**Subtype 1b (Responders vs.**	
**Relapsers)**		**Relapsers)**	
** Attribute**	**No. of selective**	**Attribute**	**No. of selective**
	**attribute**		**attribute**
	**weightings**		**weightings**
	**(out of 10)**		**(out of 10)**
Count of oxygen	10	**Count of UU**	6
**Count of UU**	7	Count of CA	5
Count of Uracil	7	Count of carbon	5
Count of nitrogen	6		

Ten algorithms (PCA, SVM, Relief, Uncertainty, Gini Index, Chi Squared, Deviation, Rule, Information Gain, and Information Gain Ratio) were used to determine the most important nucleotide attributes for the prediction of HCV subtypes 1a and 1b responders from non-responders (A) and responders from relapsers (B). Common nucleotide attributes used for genotypes 1a and 1b have been bolded. A: adenine, T: thymine, C: cytosine, G: guanine.

### Trees induction

**Figure 2:**
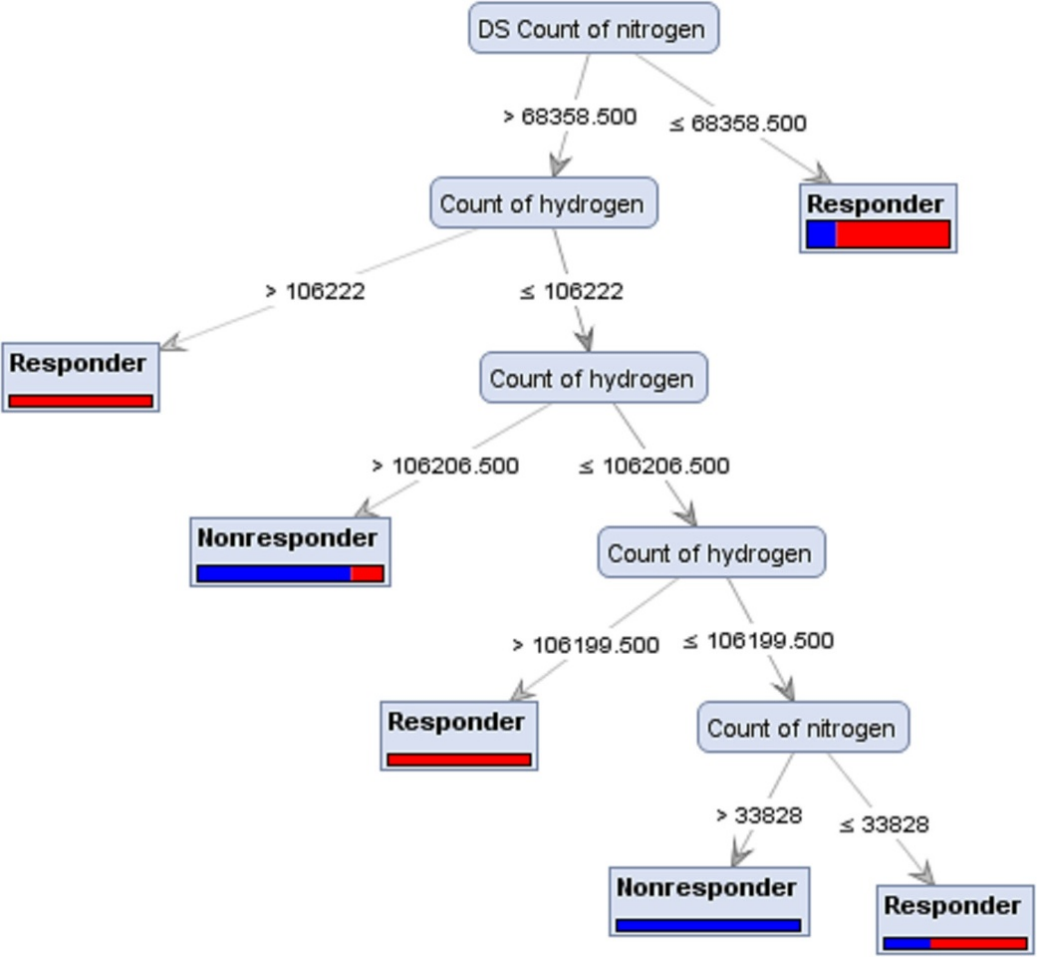
**Achieved Decision Tree from Parallel model ran with Gini Index criterion on PCA dataset, which distinguish HCV subtype 1a responders’ strains from non-responders strains.**

**Figure 3:**
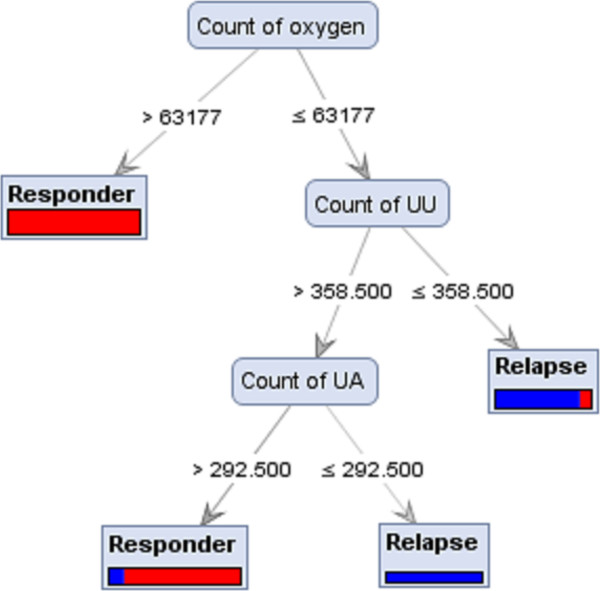
**Achieved Decision Tree from Parallel model ran with Gini Index criterion on Chi Squared dataset, which can distinguish HCV subtype 1a responder strains from relapser strains.**

**Figure 4:**
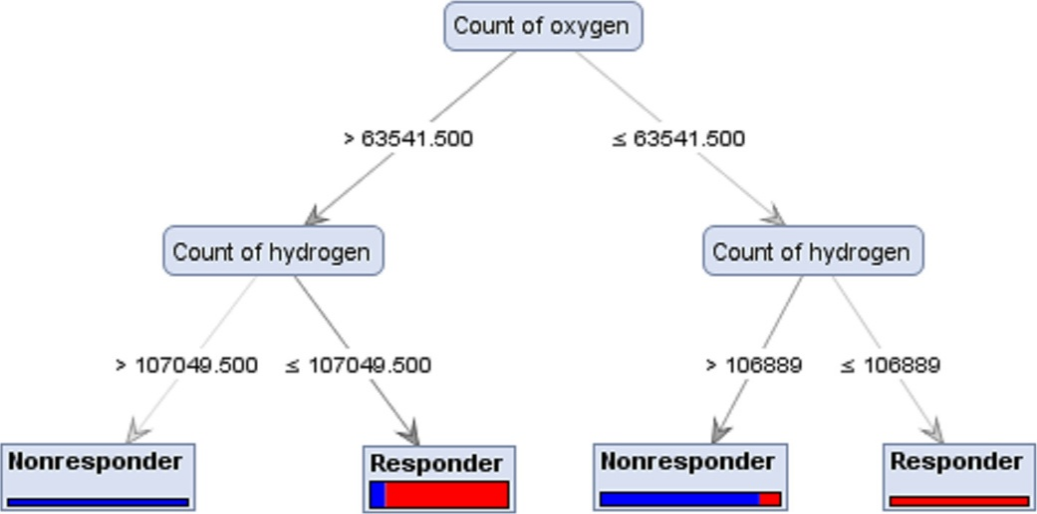
**Achieved Decision Tree model ran with Gini Index criterion on PCA Dataset, which can distinguish HCV subtype 1b responder strains from non-responder strain.**

**Figure 5:**
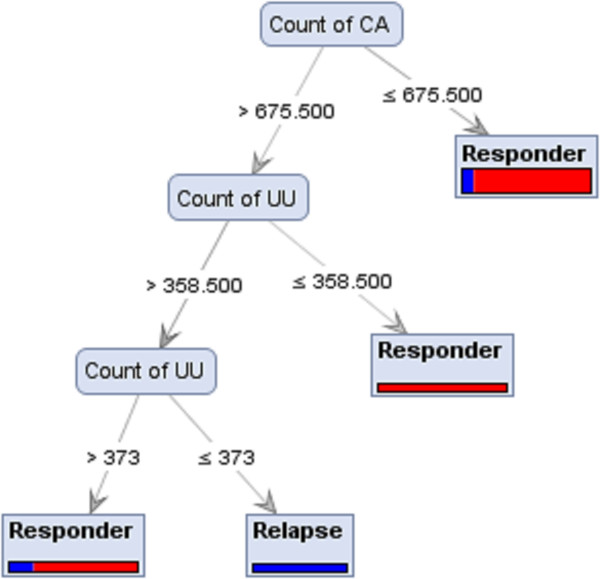
**Decision Tree from Parallel model ran with Accuracy criterion on Rule Dataset of HCV subtype 1b responders vs. relapser strains.**

A decision tree is constructed by looking for regularities in data, determining the features to add at the next level of the tree using an entropy calculation, and then choosing the feature that minimizes the entropy impurity [24]. As shown in Figure 2, a tree model for subtype 1a responders vs. non-responders was generated based on the double strand count of nitrogen and hydrogen. A nitrogen value ≤ 68358.50 classified some sequences as responders though it did not distinguish all responders. When a nitrogen value is ≥68358.50, therapy outcome depends on the hydrogen count value. Figure 3 is a tree model built on the analysis of subtype 1a responders vs. relapsers’ sequences detected the oxygen count as the root of the tree and sequences with a value >63177 were identified as responder group. When the oxygen value was <63177, the therapy outcome depends on the count of UU. Figures 4 and 5 represent the tree models developed for subtype 1b responders vs. non-responders and responders vs. relapsers’ sequences, respectively.

### Prediction algorithms

Various machine based learning algorithms [decision tree, Support Vector Machine (SVM), Naive Bayes and Neural Networks] were trained and tested to predict IFN/RBV therapy respond based on computed features. When an algorithm gained the highest accuracy it was used as fundamental predictor for IFN/RBV response treatments.

### Decision tree

Decision tree classifiers are the most popular supervised learning methods for data exploration. The trees summarize and transform data into a more compact forms that maintain the essential characteristics for an easy interpretation [25].

The best performance among 176 decision tree models (16 models run on 11 datasets) in the prediction of therapy response are presented in Table 2A and B. The highest accuracy values for the prediction of subtypes 1a and 1b responders from non-responders were 69.17% and 80.00%, respectively. The highest accuracy (81.67%) was achieved for both subtypes when responder and relapser groups were analysed.

**Table 2:** **The highest values for accuracy, AUC, F-measure, precision, recall, sensitivity, and specificity for predicting responders vs. non-responders (A) and responders vs. relapsers (B) groups**

A								
	Subtype 1a (Responders vs. Non-Responders)				Subtype 1b (Responders vs. Non-Responders)			
	Bayes	Neural Networks	SVM	Decision Trees	Bayes	Neural Networks	SVM	Decision Trees
**Database**	Chi Squared	SVM	Relief	PCA	SVM	SVM	Relief	Gini Index
**Algorithm**	Naive Bayes (Kernel)	AutoMLp	SVM	DT Parallel Gini Index	Naive Bayes (Kernel)	AutoMLp	SVM	DT Random Forest Info Gain
**Accuracy**	74.17%	76.67%	74.17%	69.17%	89.17%	85.00%	75.00%	80.00%
**AUC**	0.84	0.68	0.75	0.59	0.94	0.94	0.84	0.83
**AUC (optimistic)**	0.84	0.68	0.75	0.83	0.94	0.94	0.84	0.85
**AUC (pessimistic)**	0.84	0.68	0.75	0.58	0.94	0.94	0.84	0.80
**F-Measure**	0.78	0.82	0.80	0.73	0.92	0.87	0.80	0.86
**Precision**	0.84	0.82	0.80	0.80	0.93	0.94	0.81	0.87
**Recall**	0.73	0.82	0.88	0.73	0.93	0.83	0.83	0.90
**Sensitivity**	0.73	0.82	0.88	0.73	0.93	0.83	0.83	0.90
**Specificity**	0.85	0.75	0.60	0.65	0.85	0.80	0.50	0.65
**B**								
	**Subtype 1a (Responders vs. Relapsers)**				**Subtype 1b (Responders vs. Relapsers)**			
	**Bayes**	**Neural Networks**	**SVM**	**Decision Trees**	**Bayes**	**Neural Networks**	**SVM**	**Decision Trees**
**Database**	Chi Squared	SVM	Relief	PCA	SVM	SVM	Relief	Gini Index
**Algorithm**	Naive Bayes (Kernel)	AutoMLp	SVM	DT Parallel Gini Index	Naive Bayes (Kernel)	AutoMLp	SVM	DT Random Forest Info Gain
**Accuracy**	82.50%	79.17%	82.50%	81.67%	78.33%	78.33%	84.17%	81.67%
**AUC**	0.89	0.79	0.82	0.61		0.00		0.66
**AUC (optimistic)**	0.89	0.79	0.82	0.91		0.85		0.85
**AUC (pessimistic)**	0.89	0.79	0.82	0.74		0.15		0.47
**F-Measure**	0.84	0.86	0.86	0.84	0.87	0.87	0.91	0.89
**Precision**	0.92	0.83	0.90	0.90	0.78	0.78	0.84	0.82
**Recall**	0.82	0.92	0.87	0.80	1.00	1.00	1.00	1.00
**Sensitivity**	0.82	0.92	0.87	0.80	1.00	1.00	1.00	1.00
**Specificity**	0.85	0.55	0.75	0.85	0.00	0.00	0.29	0.14

### SVM approach

SVM algorithms have become very popular as a high-performance classifier in several fields including bioinformatics. The main objective is to construct a hyper-plane as the decision surface so that the margin of separation between positive and negative examples is maximized [25].

The best accuracy values for the 1a and 1b responder vs. non-responder sequences using SVM analysis showed 74.17% and 75.00% accuracies, respectively. SVM analysis revealed higher accuracies up to 82.50% and 84.17% for responder vs. relapser of subtypes 1a and 1b sequences, respectively. A summary of results including sensitivity, specificity, precision, recall and F-measure have been provided in Additional files 2 and 3.

For SVM analysis on the 1a and 1b responder vs. non-responder sequences, the best accuracy values were 74.17% and 75.00%, respectively. The accuracies of our approaches were estimated to be higher when responder vs. relapser of subtype 1a sequences (82.50%) and responder vs. relapser of subtype 1b sequences (84.17%) were analyzed. Accuracy values and other parameters (sensitivity, specificity, precision, recall and F-measure) have been presented in Additional files 2 and 3.

### Naïve Bayes

The Naive Bayes classifier is a simple learning algorithm that is used for data mining applications. This algorithm can also be used as a predictive model.

Analysing subtypes 1a and 1b responder vs. non-responder sequences, Naive Bayes achieved the highest accuracy of 74.17% and 89.17%, respectively. The highest accuracy for responder vs. relapser sequences were 82.50% when Bayes kernel was ran on Relief and 78.33%, when Bayes kernel was ran on Chi Squared dataset (Table 2A and B). Full results from Bayesian algorithms are presented in Additional files 2 and 3.

### Neural networks

Neural networks are computational models that are capable of machine learning and pattern recognition and have been used as predictive models.

Analyzing subtypes 1a and 1b responder vs. non-responder sequences, the Auto MLP showed the best performance with 76.67% and 85.00% accuracy, respectively, when it was ran on SVM dataset. By analyzing responder and relapser sequences, the best accuracy values were 79.17% and 78.33% for subtypes 1a and 1b, respectively (Table 2A and B). The full results of neural networks are presented in Additional files 2 and 3.

## Discussion and Conclusion

In the absence of an effective vaccine, treatment of HCV-infected patients is the only means to combat the disease. Until recently, IFN/RBV combination therapy has been commonly used to treat HCV patients. The introduction of replicon systems [26, 27] encoding enzymatically functional viral proteins such as NS3 and NS5B has led to the discovery of new anti-HCV drugs including the two NS3 inhibitors, boceprevir and telaprovir. However, the new drugs result in an increase in therapy response when they are combined with IFN and RBV [5, 28]. Several reports indicated that viral and host factors influence the combination therapy outcome. Pascu et al. [15] analysed three HCV genotype 1b genomes and identified a number of mutations in a specific region in HCV NS5A named interferon sensitivity determining region (ISDR) that predicted therapy response. Following this report, other research groups identified specific regions in other HCV proteins that predicted treatment outcome [7–9, 14, 19]. The current study aimed to analyse nucleotide sequences of HCV isolates from responders, non-responders, and relapsers aiming to generate decision trees for the prediction of treatment outcome. Recently, we have shown that the comparison of large numbers of sequences using mining techniques (such as decision tree) generated supervised and unsupervised models suitable for identification of novel proteins involved in the malignancy of breast cancer and lung cancer [29, 30] and genetic markers for characterization of olive cultivars [20]. Using the same approach, we identified new genetic determinants that play important functional roles in the thermostable proteins [31], halostable proteins [32], and P1B-ATPase heavy metal transporters [21]. Furthermore, feature selection techniques and other learning methods such as bipartite learning graph and semi-supervised algorithms have already been used in drug-target interactions and the capability of these methods in predicting drug-target datasets has been proven [33]. In the present study, we used the same strategy to evaluate correlation between HCV gene attributes at nucleotide levels with treatment response. Different attribute weighting systems used unique patterns to define the most important attributes for classification of data. To assess the genetic markers that affect the response to treatment, we analysed sequences from the patients that responded (more than 3.4 logs decline in viral load) or failed to respond (less than 1.4 log decline in viral load) to therapy at 28 days post-treatment. Analysing both subtypes 1a and 1b, some attribute weighting algorithms predicted treatment response based on the UA count (Table 1A).The nucleotide composition of viral RNA may affect its sensitivity to IFN-stimulated genes (ISGs) such as RNase L which cleaves the RNA at dinucleotides UU and UA [34]. Interestingly, dinucleotide UU was selected by 60-70% models to distinguish responders from relapsers in both subtypes. Collectively, our analyses highlight the importance of dinucleotides UA and UU in combination treatment outcome. Strikingly, count of oxygen was an important attribute only when subtype 1a responders were compared to non-responders and relapsers (Table 1A and B). Our analysis identified several other dinucleotides that were selected as determining attributes for distinguishing responders from non-responders and relapsers. These attributes may contribute to viral RNA structures that promote/prevent interactions with ISGs; however further investigation is required to determine their significance and precise role in HCV therapy response. The method employed in this study can be used to explore other viral features such as amino acids compositions, mRNA, miRNA and protein features in addition to nucleotide features.

Several mechanisms of action have been described for nucleotide analogue RBV but the exact mechanism are not fully understood [35]. Using an in vitro assay, Maag et al. [36] demonstrated that only high concentrations of RBV lead to its incorporation into HCV genome opposite of cytosine and uridine and terminated elongation process mediated by viral polymerase NS5B. Analysing subtypes 1a and 1b responders and non-responders revealed that count of guanine was selected as an important attribute only in subtype 1a sequences. However, the accounts of guanine and adenine in viral genome are not common selected determinant between both subtypes and do not support the proposed above mechanism of action of RBV at physiological concentrations. In line with our finding, it has been reported that using nucleotide analogues for treatment of HIV infections does not select for the nucleotide compositions of the viral genome [37].

The majority of induction tree models failed to distinguish between responders and relapsers and non-responders. Decision Tree and Decision Tree parallel models generated decision trees with a reasonably high accuracy for the prediction of therapy response. Some tree induction models had simple configuration with three or four branches (Figure 4) however the depth of trees in some models were more complicated (Figure 2). The highest accuracies values for the prediction of subtypes 1a and 1b responders from non-responders were 69.17% and 80.00%, respectively. In addition, the sensitivity and specificity of these models were 0.88 and 0.60 for subtype 1a and 0.90 and 0.65 for subtype 1b. When responder and relapser groups were analysed the highest accuracy, 81.67%, was achieved for both subtypes 1a and 1b and the sensitivity and specificity of these models reached up to 0.80 and 0.85 for subtype 1a and 1.00 and 0.14 for subtype 1b, respectively (Table 2A and B).

The prediction performances of Neural Net algorithm in analyzing responders vs. non-responders sequences showed that AutoMLp algorithm could be used for prediction with accuracies up to 76.67 for subtype 1a and 85.00% for subtype 1b. Also analysing subtype 1a responders vs. relapsers sequences revealed that Neural Network algorithm had the best prediction with accuracy up to 79.17 and sensitivity and specificity as high as 0.92 and 0.55, respectively; while in analyzing subtype 1b the Perceptron algorithm showed the highest accuracy with 78.33% and its sensitivity was 1.00 though its specificity was far low (Table 2A and B).

SVM is a supervised non-parametric statistical learning technique, therefore there is no assumption problems usually involve identification of multiple classes. From seven models of SVM applied on 11 datasets of subtypes 1a and 1b in this study, the accuracies of SVMs algorithm were high (74.14% for 1a and 75.00% for 1b) for responders vs. non-responders sequences in both subtypes. By comparing responders vs. relapsers sequences, SVM algorithm showed 82.50% accuracy for subtype 1a while SVM LibSVM demonstrate the highest accuracy (84.17%) for subtype 1b (Table 2A and B).

When Naïve Bayes models trained with machine learning models, the performances of Naïve Bayes Kernel models were generally higher than models ran with Naïve Bayes (without Kernel). The highest possible accuracies gained when Naïve kernel Base models ran on the responders vs. non-responders sequences (74.17% and 89.17% for subtypes 1a and 1 b, respectively) and the sensitivity and specificity reached up to 0.73 and 0.85 for subtype 1a, and 0.93 and 0.85 for subtype 1b. These achieved criteria make Naïve Bayes Kernel one of the best prediction models in comparing responders vs. non-responders sequences. Our findings suggested that Naïve-Based Kernel model has the best prediction accuracy in comparing responders vs. relapsers’ sequences with figures up to 82.50% and 78.33% for subtypes 1a and 1b, respectively. Therefore, this model could be regarded as the most suitable algorithm for prediction responders vs. relapsers’ sequences (Table 2A and B).

In conclusion, we applied combination of different algorithms on the genomic attributes of HCV subtypes 1a and 1b and identified novel genetic markers including several dinucleotides that predicted therapy response rate with high accuracy.

## Methods

Ninety three full length nucleotide sequences from subtypes 1a (12 relapsers, 22 responders and 13 non-responders) and 1b (7 relapsers, 26 responders and 13 non-responders) [14, 19] were extracted from GenBank. Further information regarding the outcome of the treatment was extracted from Virahep study (publically available) by Dr Donlin, Department of Biochemistry & Molecular Biology, Saint Louis University School of Medicine, USA. Some original sequences contained deletions at their extreme ends. To avoid the effects of length factor of the sequences, the 93 sequences were aligned and adjusted by removing the extreme ends of all other sequences. The above sequences have been obtained by direct sequencing of PCR products from pre-treatment samples [19]. The adjusted nucleotide sequences of HCV subtypes 1a and 1b were divided into 2 groups thereby creating four datasets; responders vs. non-responders, responders vs. relapsers for each subtype. Seventy six gene attributes – e.g. count and frequency of each nucleotide, di-nucleotides and elements, and molarities of salt contents (the concentration of monovalent cations in units of molar) were extracted using various bioinformatics tools and various software including CLC bio software (CLC bio, Finlandsgade 10–12, Katrinebjerg 8200 Aarhus N Denmark). List of each attributes and calculated values are presented in Additional files 4 and 5. Treatment types feature were categorical, and the other attributes were continuous variables. A dataset of these gene attributes was imported into Rapid Miner software [RapidMiner 5.0.001, Rapid-I GmbH, Stochumer Str. 475, 44227 Dortmund, Germany]. The null data for treatment type attribute was excluded, and this attribute was set as the output variable and the other variables were set as input variables.

### Data filtering

We did not detect any duplicated attributes (two examples were assumed equal if all values of all selected attributes were equal) in our datasets. Related (with Pearson correlation greater than 0.9) and useless attributes were excluded from the dataset. Numerical attributes which possessed standard deviations less than or equal to a given deviation threshold (0.1) were considered useless. The final dataset was named final cleaned database (FCdb).

### Attribute weighting

To identify the most important gene attributes, and to find likely patterns in ones that contribute to HCV therapy responses, 10 different algorithms of attribute weightings namely Information Gain, Information Gain Ratio, Rule, Deviation, Chi Squared, Gini Index, Uncertainty, Relief, Support Vector Machine (SVM) and PCA were applied to the FCbd as described previously [22, 23, 38].

Following attribute weighting application, each attribute gained a value between 0–1 indicating the importance of it. All variables with weights equal to or higher than 0.5 selected and saved as new dataset; consequently, 10 new datasets were created. These newly formed datasets named according to their applied attribute weighting models (Information gain, Information gain ratio, Rule, Deviation, Chi Squared, Gini index, Uncertainty, Relief, SVM and PCA).

### Machine learning algorithms

Three classes of machine learning algorithms namely Trees Inductions, SVM, and Bayesian along with the Neural Network were used to analyze the 11 datasets (see above) and construct models suitable for the prediction of HCV therapy response. To calculate the performance of each algorithm, 10-fold cross validation [39] was used to train and test models on all patterns. The classifier was trained on 90% of the data, and the remaining 10% were used as an unseen test set to assess the classifier’s performance. This procedure was repeated 10 times (10-folds), with a different 10% of the data randomly selected as the test set in each repeat [40, 41]. In this study, accuracy is calculated by taking the percentage of correct predictions over the total number of examples. Correct prediction means examples where value of prediction attribute is equal to the value of label attribute.

### Trees induction algorithms

Four Trees Induction algorithms namely Decision Tree, Decision Tree Parallel, Decision Stump and Random Forest were run on the 11 datasets described in attribute weighting section. Each tree induction algorithms ran with Gain Ratio, Information Gain, and Gini Index and accuracy criteria. All algorithms except Random Forest created only one decision tree model; on the other hand Random Forest algorithm generated 10 different trees models for each criteria and consequently 572 trees were generated by all algorithms [38]. All models were trained and tested with 10-fold cross validation and the averages of the accuracy values were calculated.

### SVMs

SVMs are theoretically well-established intuitive and feasible techniques for classification and prediction of supervised data [42–47]. We used seven SVMs models to classify and predict the HCV treatment response.

SVM learner uses the Java implementation of the support vector machine mySVM by Stefan Rueping [48]. This learning method can be used for both regression and classification and provides a fast algorithm and good results for many learning tasks. mySVM works with linear or quadratic and even asymmetric loss functions. This algorithm used dot kernel type for its learning process.

LIBSVM is an integrated software for support vector classification, (C-SVC, nu-SVC), regression (epsilon-SVR, nu-SVR) and distribution estimation (one-class SVM). It supports multi-class classification. The kernel type used for this algorithm was rbf.

Fast Large Margin learner is based on the linear support vector-learning scheme proposed by R.-E. Fan, K.-W.Chang, C.-J.Hsieh, X.-R.Wang, and C.-J. Lin. Although its results is similar to those delivered by classical SVM or logistic regression implementations, this linear classifier is able to work on data set with millions of examples and attributes.

Linear SVM is an extremely fast machine learning (data mining) algorithm for solving multi-class classification problems from ultra large data sets that implements an original proprietary version of a cutting plane algorithm for designing a linear SVM. Linear SVM is a linearly scalable routine meaning that it creates an SVM model in a CPU time, which scales linearly with the size of the training data set.

Evolutionary SVM (ESVMs) incorporate the learning engine of SVMs but develop the coefficients of the decision function by means of evolutionary algorithms. This algorithm used radial kernel type in this study.

SVMPSO is initialized with a group of random particles (solutions) and then searches for most efficient particles by updating each generation [49]. This algorithm trained with radial kernel type.

Hyper SVM model identifies the best parameter orders to reproduce a new classification. This model utilizes a Huffman-Tree like mechanism, called hyperSVM [50]. Briefly, main database (FCdb) transformed to SVM format and scaled by grid search (to avoid attributes in greater numeric ranges dominating those in smaller numeric ranges) and to find the optimal values for operator parameters. Datasets were divided into 10 subsets; 9 training subsets and 1 testing subset.

To ensure the best performance of SVM models, different parameters including accuracies, specificity, sensitivity, F-measure, and AUC (pessimist and optimist) were calculated.

### Naïve Bayes

Naïve Bayes is based on the Bayes conditional probability Rule and represents an attractive classification tool when predictors are statistically independent. Two classification models namely Naïve base and Naïve base kernel trained with 10-fold cross validation on all 11 datasets to predict responders/relapsers and non-responders. The accuracy of models was estimated as described [29].

### Neural network

Two neural net algorithms (Neural Net and AutoMLP) trained with 10-fold cross validation on all 11 databases and the model accuracies in predicting the right protein’s class computed as stated before.

Neural Net is a procedure that trains a neural net using a feed-forward neural network algorithm. A feed-forward network is an artificial neural network with an algorithm where the connections between the modeled neurons are such that the information propagation is in one direction, from the input node, through intermediate nodes, to the output node.

A multilayer perceptron (MLP) is a feed-forward artificial neural network model that maps sets of input data on a set of suitable output. An MLP contains of multiple layers of nodes in a directed graph. Except for the input nodes, each node is a processing element that has a nonlinear activation function. MLP employs back propagation for training the network. This class of networks includes multiple layers of computational elements, usually interconnected in a feed-forward way. In many applications, the units of these networks apply a sigmoid function as an activation function.

In this study, a default-hidden layer with sigmoid type and size (number of attributes + number of classes)/2 + 1 created and added to the net and the training cycle was set to 500. The used activation function was the usual sigmoid function. Therefore, the values ranges of the attributes scaled to −1 and +1. The type of the output node is sigmoid because the learning data described a classification task.

AutoMLP algorithm combines ideas from genetic algorithms as well as stochastic optimization [51]. The method maintains a small ensemble of networks that are trained in parallel with different rates and different numbers of hidden units. After a small, fixed number of epochs, the error rate is determined on a validation set and the worst performers are replaced with copies of the best networks, modified to have different numbers of hidden units and learning rates. Hidden unit numbers and learning rates are drawn according to probability distributions derived from successful rates and sizes.

## Electronic supplementary material

Additional file 1: **The attribute weighting results of 1a and 1b virus subtypes.** (ZIP 29 KB)

Additional file 2: **The prediction performances of 1b virus subtype treatment groups.** (ZIP 61 KB)

Additional file 3: **The prediction performances of 1a virus subtype treatment groups.** (ZIP 57 KB)

Additional file 4: **List of calculated attributes for each sequence.** (DOCX 20 KB)

Additional file 5: **The initial created datasets of 1a and 1b virus subtypes.** (ZIP 59 KB)
