# Supplementary material for: Prediction of hepatitis C virus interferon/ribavirin therapy outcome based on viral nucleotide attributes using machine learning algorithms
Source: BMC Res Notes. 2014 Aug 23;7:565. doi: 10.1186/1756-0500-7-565 (PMC4246553; doi:10.1186/1756-0500-7-565)
Supplement: Supplementary file 4 — Additional file 4: List of calculated attributes for each sequence. (DOCX 20 KB) [file 13104_2014_3338_MOESM4_ESM.docx]

| List of calculated attributes for each sequence | | | | | | | |
| --- | --- | --- | --- | --- | --- | --- | --- |
| Length | Frequency of CG | Count of UA | Count of AG | Frequency of Guanine | DS Frequency of oxygen | Frequency of oxygen | Count of nitrogen |
| Weight | Frequency of CU | Count of UC | Count of AU | Frequency of Uracil | DS Frequency of phosphorus | Frequency of phosphorus | Count of oxygen |
| DS Weight | Frequency of GA | Count of UG | Count of CA | Frequency of C + G | Count of Adenine | DS Count of hydrogen | Count of phosphorus |
| salt 0.1M | Frequency of GC | Count of UU | Count of CC | Frequency of A + U | Count of Cytosine | DS Count of carbon | Frequency of hydrogen |
| salt 0.2M | Frequency of GG | Frequency of AA | Count of CG | Percentage of A | Count of Guanine | DS Count of nitrogen | Frequency of carbon |
| salt 0.3M | Frequency of GU | Frequency of AC | Count of CU | Percentage of C | Count of Uracil | DS Count of oxygen | Frequency of nitrogen |
| salt 0.4M | Frequency of UA | Frequency of AG | Count of GA | Percentage of G | Count of C + G | DS Count of phosphorus |  |
| salt 0.5M | Frequency of UC | Frequency of AU | Count of GC | Percentage of U | Count of A + U | DS Frequency of hydrogen |  |
| Count of hydrogen | Frequency of UG | Frequency of CA | Count of GG | Count of AA | Frequency of Adenine | DS Frequency of carbon |  |
| Count of carbon | Frequency of UU | Frequency of CC | Count of GU | Count of AC | Frequency of Cytosine | DS Frequency of nitrogen |  |
